# Efficacy of rhBMP-2 Loaded PCL/*β*-TCP/bdECM Scaffold Fabricated by 3D Printing Technology on Bone Regeneration

**DOI:** 10.1155/2018/2876135

**Published:** 2018-02-27

**Authors:** Eun-Bin Bae, Keun-Ho Park, Jin-Hyung Shim, Ho-Yun Chung, Jae-Won Choi, Jin-Ju Lee, Chang-Hwan Kim, Ho-Jun Jeon, Seong-Soo Kang, Jung-Bo Huh

**Affiliations:** ^1^Department of Prosthodontics, Dental Research Institute, Institute of Translational Dental Sciences, BK21 PLUS Project, School of Dentistry, Pusan National University, Yangsan 50612, Republic of Korea; ^2^Department of Mechanical Engineering, Korea Polytechnic University, Siheung 15073, Republic of Korea; ^3^Department of Plastic and Reconstructive Surgery, School of Medicine, Kyungpook National University, Daegu 41944, Republic of Korea; ^4^Research Institute, T&R Biofab Co., Ltd., Siheung 15073, Republic of Korea; ^5^Department of Veterinary Surgery, College of Veterinary Medicine, Chonnam National University, Gwangju 61186, Republic of Korea

## Abstract

This study was undertaken to evaluate the effect of 3D printed polycaprolactone (PCL)/*β*-tricalcium phosphate (*β*-TCP) scaffold containing bone demineralized and decellularized extracellular matrix (bdECM) and human recombinant bone morphogenetic protein-2 (rhBMP-2) on bone regeneration. Scaffolds were divided into PCL/*β*-TCP, PCL/*β*-TCP/bdECM, and PCL/*β*-TCP/bdECM/BMP groups. In vitro release kinetics of rhBMP-2 were determined with respect to cell proliferation and osteogenic differentiation. These three reconstructive materials were implanted into 8 mm diameter calvarial bone defect in male Sprague-Dawley rats. Animals were sacrificed four weeks after implantation for micro-CT, histologic, and histomorphometric analyses. The findings obtained were used to calculate new bone volumes (mm^3^) and new bone areas (%). Excellent cell bioactivity was observed in the PCL/*β*-TCP/bdECM and PCL/*β*-TCP/bdECM/BMP groups, and new bone volume and area were significantly higher in the PCL/*β*-TCP/bdECM/BMP group than in the other groups (*p* < .05). Within the limitations of this study, bdECM printed PCL/*β*-TCP scaffolds can reproduce microenvironment for cells and promote adhering and proliferating the cells onto scaffolds. Furthermore, in the rat calvarial defect model, the scaffold which printed rhBMP-2 loaded bdECM stably carries rhBMP-2 and enhances bone regeneration confirming the possibility of bdECM as rhBMP-2 carrier.

## 1. Introduction

Scaffolds used for bone tissue regeneration should be biocompatible and biodegradable and have appropriate mechanical properties and architectures [1]. The evolution of 3D printing technology means that the required scaffold characteristics, such as shape, surface morphology, and pore shapes and dimensions, have become more accessible [2]. 3D printing can be used to produce specific shapes easily, quickly, and reproducibly, and many attempts have been made to use 3D printing technology in the tissue engineering field [2, 3].

Synthetic bioresorbable polymers like polycaprolactone (PCL), polyglycolide (PGA), polylactide (PLA), and poly lactic-co-glycolic acid (PLGA) have been used to produce 3D printed scaffolds [4–8] but are limited in terms of stem cell attachment and osteogenic differentiation [8]. Because it has proven to be difficult to produce bone grafts or scaffolds that can achieve satisfactory bone regeneration results using available synthetic polymers, several efforts have been made to incorporate bioactive materials into synthetic polymers [7, 9, 10].

Natural extracellular matrix (ECM) contains collagens, noncollagenous proteins (NCPs), and proteoglycans, which all play important roles in cell proliferation and differentiation by providing microenvironments with suitable intercellular connections [11]. It has been found difficult to develop biomaterials by mimicking the ECM compositions of target tissues [11, 12]. For this reason, in the bone tissue engineering field, demineralized bone matrix (DBM) produced using ECM extracted from bovine bone has been widely used [13, 14]. DBM reproduces the microenvironment as it contains growth factors, collagen, and NCPs [15] and, thus, is osteoconductive and osteoinductive [16]. In order to suppress immune response to DBM xenografts, a bone demineralized and decellularized extracellular matrix (bdECM) gel has been used [17, 18]. In a recent study, 3D printing of bdECM on PCL/PLCA/*β*-TCP porous scaffolds was found to promote bone regeneration and improve osteoblast adhesion and proliferation [18]. Interestingly, it was suggested that bdECM could be used as a bioink for bioprinting cells [12, 18], although in a subsequent study, it was found difficult to maintain high cell density and vitality during the fabrication and transplantation process and to reconstruct small dental defects [19].

In the dental field, osteoinductive proteins, such as members of the recombinant human bone morphogenetic protein (BMP) family, are widely used to increase bone regeneration [20]. BMP promotes mesenchymal stem cell (MSC) proliferation and differentiation into osteoblasts, which promote new bone formation [20, 21]. rhBMP-2 is the most osteoinductive member of the BMP family [22] and is being applied clinically for maxillary sinus lift [23] and alveolar bone augmentation [24]. However, rhBMP-2 is lost rapidly after application and has side effects when overdosed [25] and, thus, various methods of fixing and transporting rhBMP-2 have been studied [26, 27].

Several studies have used various carriers to bind rhBMP-2 to the bone grafts but did not achieve a satisfactory rhBMP-2 release and bone regeneration. bdECM which already contains a small amount of rhBMP-2 is a promising carrier for growth factor proteins, but there has been no attempt to use bdECM to deliver rhBMP-2. Therefore, the present study was undertaken to evaluate the possibility of using bdECM as rhBMP-2 carrier and the effects of 3D printed PCL/*β*-TCP scaffolds in combination with rhBMP-2 loaded bdECM on osteogenesis in a rat calvarial defect model.

## 2. Materials and Methods

### 2.1. Preparation of Scaffold for Analysis

#### 2.1.1. Preparation of bdECM

Porcine bone was used to prepare bdECM. Soft tissue and marrow of the porcine bone were manually removed with using surgical blade, and the remaining bone was freeze-dried at −85°C for 24 hr and ground to size particles of porcine bone (SPB). The SPB obtained was then washed with 70% ethanol to remove fat and demineralized in 0.5 N hydrochloric acid (HCL), and HCL solution was replaced every 2 hr to remove undesirable particles 3 times. The demineralized SPB was then washed with distilled-deionized water (DW) 3 times, placed in a solution containing 0.05% trypsin and 0.02% EDTA for 2 hr at 37°C, washed with DW 3 times, freeze-dried at −85°C for 24 hr, and powdered in a freezer mill (6875D, SPEX SamplePrep, Metuchen, NJ, USA). The powdered porcine bone (PB) obtained was then solubilized using an acidic pepsin solution to obtain bdECM gel.

#### 2.1.2. Preparation of bdECM Containing rhBMP-2

rhBMP-2 (Cowellmedi Co., Ltd., Pusan, South Korea) and bdECM were blended by mixing as follows. The gel-state bdECM (0.9 ml) was placed in plastic container and maintained at 4°C. Separately, powdered rhBMP-2 was dissolved in DW using a vortex mixer (G560E, Scientific Industry Inc., NY, USA), respectively. rhBMP-2 solutions were added to bdECM and manually mixed for 3 min.

#### 2.1.3. Preparation of Blended PCL/*β*-TCP

PCL (PC 12, Corbion Purac, Gorinchem, Netherlands) and *β*-TCP (7758-87-4, Premier Biomaterials, Tipperary, Ireland) were blended together using a melt process. PCL (7 g) was placed in glass container and melted by heating for 15 min at 110°C. Powdered *β*-TCP (3 g) was then added and the mix was blended by hand for 10 min.

#### 2.1.4. Fabrication of Experimental Scaffolds

**Figure 1:**
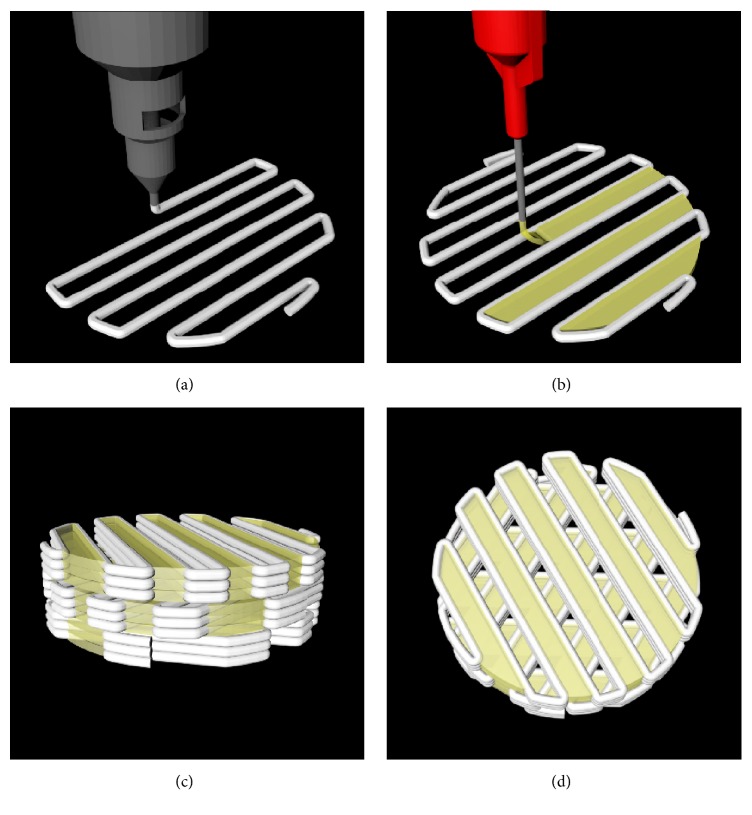
Graphical images of (a, b) scaffold fabricating procedure and (c, d) final design. (a) Printing of blended PCL/*β*-TCP. (b) Printing of bdECM into the gap between the printed lines of PCL/*β*-TCP. (c) Side view and (d) top view of the designed scaffold containing bdECM.

**Figure 2:**
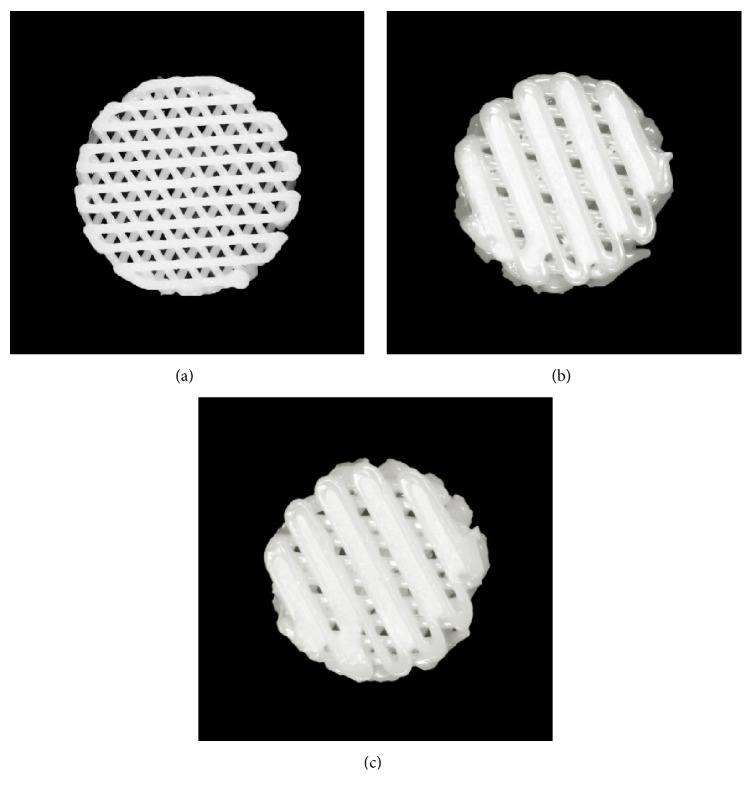
Fabricated scaffolds used in the (a) PCL/*β*-TCP, (b) PCL/*β*-TCP/bdECM, and (c) PCL/*β*-TCP/bdECM/BMP groups.

PCL/*β*-TCP and bdECM with or without rhBMP-2 were placed in the 10 ml steel and 3 ml plastic syringes of a 3D printing system and maintained at 120°C and 4°C, respectively. The 3D printing system was operated using self-developed CAM software. The PCL/TCP material was extruded and stacked 3 times. After the stacking of the PCL/TCP, bdECM was dispensed into the gap between the stacked PCL/TCP (Figure 1). The scaffolds used in the in vitro study were 7 × 7 mm square and 1 mm thick. The in vivo study was conducted using 8 mm diameter circular scaffolds of 1 mm thickness. The line width, pore size, and line height of scaffolds were 300, 400, and 100 *μ*m, respectively [18]. The scaffolds had triangular pore architecture and a porosity of 57% as determined by 3D modeling software 3-Matic Research (ver. 9.0, Materialise, Leuven, Belgium). Pores were fully interconnected. The calculated volume of bdECM dispensed in scaffolds in vitro and in vivo was about 17 *μ*l and 16 *μ*l, respectively. The initial amount of rhBMP-2 loaded into each scaffold was 5 *μ*g [7]. The scaffolds were placed in an incubator at 37°C to reticulate the bdECM, freeze-dried at −85°C for 24 hours, and sterilized by being placed under a 450 W UV lamp for 4 hours. Scaffolds were divided into three groups, and the groups were as follows (Figure 2):

1. PCL/*β*-TCP: PCL (70 wt%)/*β*-TCP (30 wt%) scaffold
2. PCL/*β*-TCP/bdECM: scaffold with bdECM printed between lines of PCL/*β*-TCP
3. PCL/*β*-TCP/bdECM/BMP: scaffold with bdECM containing rhBMP-2 (5 *μ*g) printed between lines of PCL/*β*-TCP

### 2.2. In Vitro Analysis

#### 2.2.1. Release Kinetics of rhBMP-2

To study release kinetics of rhBMP-2 in the PCL/*β*-TCP/bdECM/BMP group, a scaffold and 3 ml phosphate buffer saline (PBS) were placed in each well of 6-well plate and incubated at 37°C for 1, 3, 5, 7, 14, 21, and 28 days when samples (1 ml) were collected (fresh PBS (1 ml) was added to maintain constant volume). Concentrations of rhBMP-2 in the collected PBS samples were determined using an ELISA kit (DBP200, R&D Systems, Minneapolis, MN), and cumulative amounts of rhBMP-2 released were expressed as initial amounts loaded (5 *μ*g).

#### 2.2.2. Cell Culture and Seeding of MC3T3-E1

MC3T3-E1 cells (mouse preosteoblasts) were cultured in *α*-minimum essential medium (*α*-MEM; Gibco BRL) containing 10% fetal bovine serum (FBS) and 1% penicillin/streptomycin (Gibco BRL). Cells were incubated in a humidified 5% CO_2_ atmosphere condition at 37°C and the culture medium was changed every 2 days. For the proliferation assay, cells were seeded at a density of 1 × 10^5^ cells per scaffold and cultured in the medium detailed above. For the osteogenic differentiation assay, cells were seeded at a density of 3 × 10^5^ cells per scaffold and cultured in osteogenic medium (*α*-MEM containing 20% FBS, 10-8 M dexamethasone, 0.2 mM ascorbic acid, 10 mM *β*-glycerol phosphate (Sigma Aldrich), and 1% penicillin/streptomycin).

#### 2.2.3. Analysis of Cell Proliferation and Osteogenic Differentiation

To quantify MC3T3-E1 cell proliferation on scaffolds, we used the Cell Count Kit-8 (CCK-8, Dojindo, Japan) on days 1, 3, and 7 after seeding. CCK-8 solution was diluted 1 : 10 with culture medium and added to samples, which were then incubated for 3 hr at 37°C. The optical densities of culture supernatants were then measured at 450 nm using a microplate reader (Epoch, BioTek, VT, USA). ALP activity was quantified using p-nitrophenyl phosphate (pNPP; Sigma Aldrich) on days 3, 7, and 14 after seeding. Samples were lysed (RIPA lysis buffer) and incubated in pNPP solution at 37°C for 30 min, and ALP activity was measured at 405 nm using a microplate reader and quantified using a p-nitrophenol standard. To estimate calcium deposition on scaffolds, samples were fixed with 4% paraformaldehyde and stained with 2% alizarin red solution (pH 4.2) for 10 min at room temperature on days 3, 7, and 14 after seeding. The alizarin red was extracted from samples using 10% cetylpyridinium chloride and quantified by measuring absorption at 570 nm using a microplate reader.

### 2.3. In Vivo Analysis

#### 2.3.1. Experimental Animals

Twenty-eight Sprague-Dawley rats (male, 12 weeks old, weight: 250–300 g, Koatech, Korea) were used in this experiment. Before the experiment, rats were adapted for a minimum of 7 days. Animals were housed individually in plastic cages under standard laboratory conditions (temperature: 25 ± 1°C, humidity: 55 ± 7%) and had ad libitum access to water and rodent pellets throughout the experiment. The study was performed at the Laboratory Animal Resource Center of Pusan National University and approved by the Pusan National University Institutional Animal Care and Use Committee (PNU-2016-1407).

#### 2.3.2. Surgical Procedures

**Figure 3:**
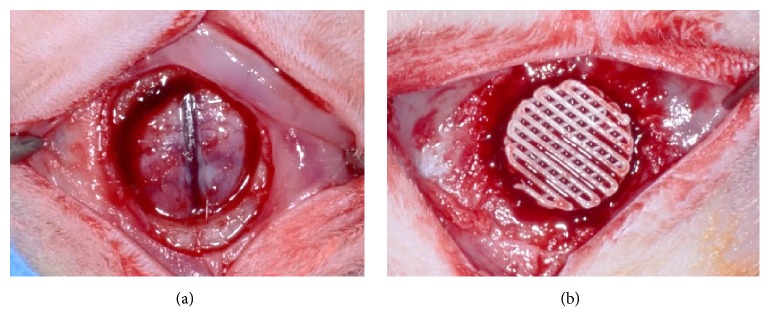
Scaffolds of each group were randomly placed in 8 mm calvaria defect. (a) Formation of 8 mm calvarial defect. (b) An experimental scaffold in situ.

All procedures were performed under general anesthesia induced by intramuscular injection with a mixture of xylazine (Rompun, Bayer Korea, Seoul, Korea) and tiletamine-zolazepam (Zoletil 50®, Virbac Laboratories, Carros, France). Surgical sites were shaved and disinfected with povidone-iodine (Betadine, Korea Pharma Co., Seoul, Korea). Local anesthesia was performed with lidocaine (2%, 1 : 100,000 epinephrine, Yuhan, Seoul, Korea). After making an incision on the cranium at midline, skin and periosteum were elevated and a critical size bone defect (8 mm diameter) was formed in the middle of the calvaria using a trephine bur (3i Implant Innovations, Inc., USA) while irrigating with saline solution (Figure 3(a)). In a total of 28 rats used, seven rats were assigned to each of the four study groups. In the control group, no scaffold was placed. In the experimental groups, the scaffolds were randomly placed (Figure 3(b)). Periosteum and skin were sutured with absorbable sutures (4-0, Vicryl®, Ethicon, NJ, USA) and black silk (4-0, Alee Co., Korea), respectively. At 4 weeks after surgery, animals were sacrificed by CO_2_ inhalation. Defect sites were then harvested with surrounding bone, and the specimens so obtained were immersed in neutral buffered formalin (Sigma Aldrich Co., St. Louis, MO, USA) for 2 weeks.

#### 2.3.3. Microcomputed Tomography (*μ*CT) Analysis

The collected calvaria specimens were wrapped with film (Parafilm M®, Bemis Co., WI, USA) to prevent fixative solution evaporation during scans. *μ*CT scanning was performed using a *μ*CT scanner (Skyscan-1173, ver. 1.6, Bruker-CT Co., Kontich, Belgium) using the following conditions: intensity: 60 *μ*A, energy: 130 kV, and pixel resolution: 7.10 *μ*m. The Nrecon reconstruction program (ver. 1.6.10.1, Bruker-CT Co., Kontich, Belgium) was used to reconstruct images. A region of interest (ROI) of the same diameter as calvaria defects (8 mm) was established. To determine total new bone volumes (NBV; mm^3^), we measured volumes occupied by new bone in ROIs.

#### 2.3.4. Histology Analysis

**Figure 4:**
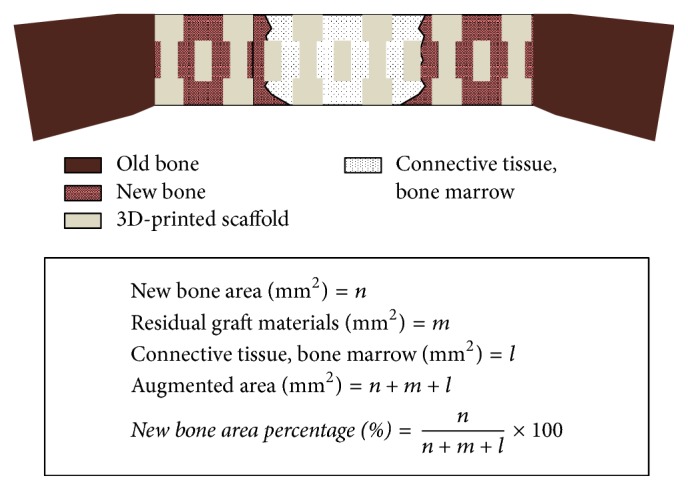
Schematic design of the histometric analysis.

The specimens were sequentially dehydrated using 70%, 80%, 90%, and 100% alcohol. After dehydration, the specimens were infiltrated with alcohol and Technovit 7200 resin (Heraeus Kulzer, Wehrheim, Germany) mixture, placed in base molds, and cured in a UV embedding system (KULZER EXAKT 520, Germany) for 12 hours. Embedded specimens were sectioned 400 *μ*m using a microtome (KULZER EXAKT 300, Norderstedt, Germany) and polished using an EXAKT grinding machine (KULZER EXAKT 400CS, Germany) to a thickness of 30 *μ*m. Slides were stained with hematoxylin-eosin staining for histologic examination and photographed using an optical microscope (BX51, OLYMPUS, Japan) equipped with a CCD camera (Polaroid DMC2 Digital Microscope Camera, Polaroid Co., MA, USA). Slides were observed at magnifications of ×12.5, ×40, and ×100. New bone areas (%) were determined using an image analysis program (i-Solution, IMT, Vancouver, Canada) by a blinded investigator (Figure 4).

### 2.4. Statistical Analysis

Results are expressed as means, standard deviations (SD), and medians. In vitro results were compared by one-way ANOVA with Tukey's post hoc test using SPSS (ver. 23, SPSS Inc., Chicago, IL, USA). In vivo results were analyzed by Brunner & Langer nonparametric analysis using Software R (ver. 3.1.3, The R Foundation, Vienna, Austria) [28]. Statistical significance was accepted for *p* values < .05.

## 3. Results 

### 3.1. In Vitro Results

#### 3.1.1. Observations of Surface Morphology

**Figure 5:**
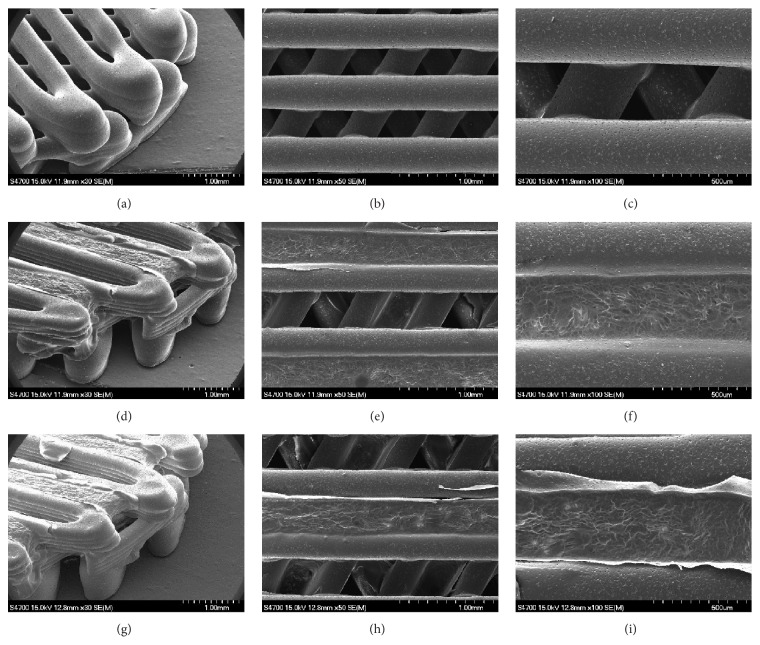
Scanning electron microscopic photographs of (a, b, and c) PCL/*β*-TCP, (d, e, and f) PCL/*β*-TCP/bdECM, and (g, h, and i) PCL/*β*-TCP/bdECM/BMP scaffolds [original magnifications: ×30 (a, d, and g) ×50 (b, e, and h), and ×100 (c, f, and i)].

SEM analysis was used to examine surface morphologies of PCL/*β*-TCP, PCL/*β*-TCP/bdECM, and PCL/*β*-TCP/bdECM/BMP scaffolds (diameter: 8 mm and height: 1 mm; Figure 5). Scaffolds had a triangular pore structure (line width: 300 *μ*m, pore size: 400 *μ*m). In the PCL/*β*-TCP/bdECM (Figures 5(d), 5(e), and 5(f)) and PCL/*β*-TCP/bdECM/BMP (Figures 5(g), 5(h), and 5(i)) groups, bdECM and rhBMP-2 loaded bdECM were dispensed, respectively, between PCL/*β*-TCP lines.

#### 3.1.2. Release of rhBMP-2 from bdECM

**Figure 6:**
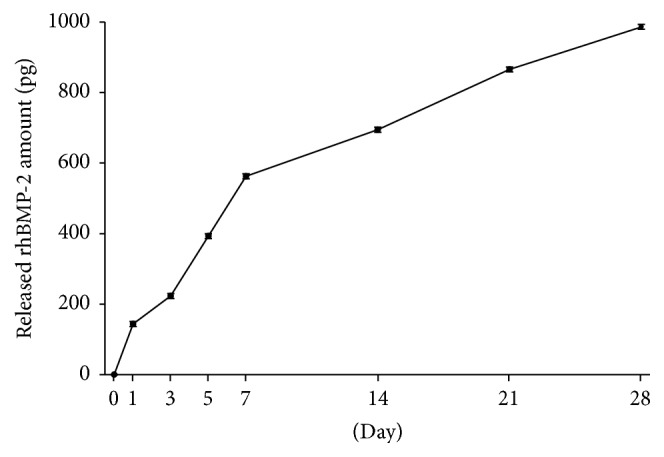
Release kinetics of rhBMP-2.

Cumulative release of rhBMP-2 from bdECM is shown in Figure 6. The amount of rhBMP-2 released is expressed as cumulative release amount (initial rhBMP-2 loading was 5 *μ*g).

#### 3.1.3. Analysis of Bioactivity

**Figure 7:**
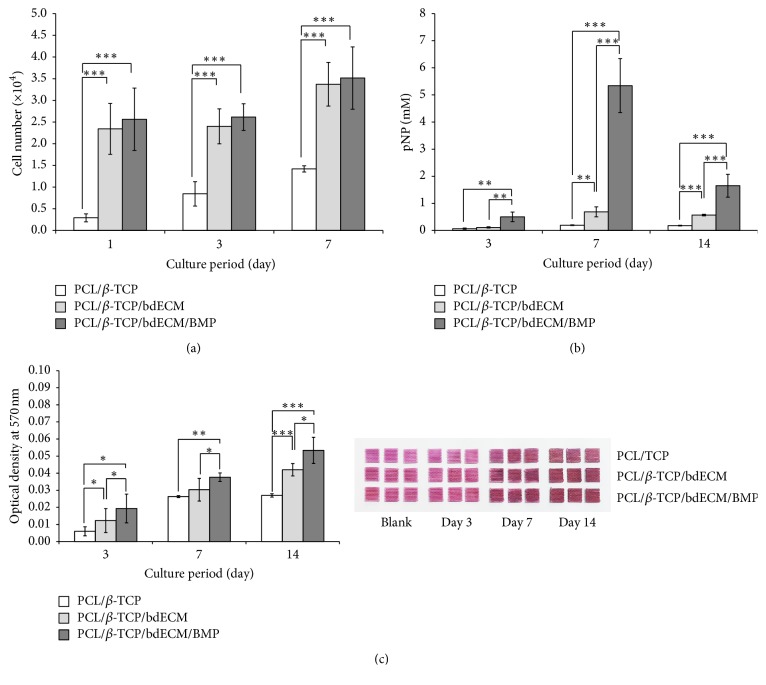
MC3T3-E1 cell proliferation and osteogenic differentiation assay. (a) Cell proliferation, (b) ALP activity, and (c) alizarin red S (^*∗*^*p* < .05, ^*∗∗*^*p* < .01, and ^*∗∗∗*^*p* < .001).

The proliferation of MC3T3-E1 cells (a mouse preosteoblast cell line) on scaffolds was examined using a CCK-8 assay (Figure 7(a)). The PCL/*β*-TCP/bdECM and PCL/*β*-TCP/bdECM/BMP groups showed higher initial cell adhesion than the PCL/*β*-TCP group after culture for 24 h, and this difference was significant at up to 7 days. The expression of alkaline phosphatase (ALP; regarded as an early marker of osteogenesis) and amounts of calcium deposited were measured to evaluate osteogenic differentiation (Figure 7(b)). In terms of ALP expression, the PCL/*β*-TCP/bdECM/BMP group showed higher ALP expression than the other groups on days 1, 3, and 7 and was greatest on day 7. Alizarin red S staining showed that the PCL/*β*-TCP/bdECM/BMP group contained more mineral calcium than the PCL/*β*-TCP and PCL/*β*-TCP/bdECM groups throughout the observation period (Figure 7(c)). Also, the amount of calcium deposition on scaffolds was greatest in PCL/*β*-TCP/bdECM/BMP group.

### 3.2. In Vivo Results

#### 3.2.1. Clinical Findings

All 28 rats survived during the procedure, and 28 defect samples were collected without any issue. During the healing period, no scaffold was exposed, and no infection or inflammation was observed at surgical sites.

#### 3.2.2. Microcomputed Tomography (*μ*CT) Findings

**Figure 8:**
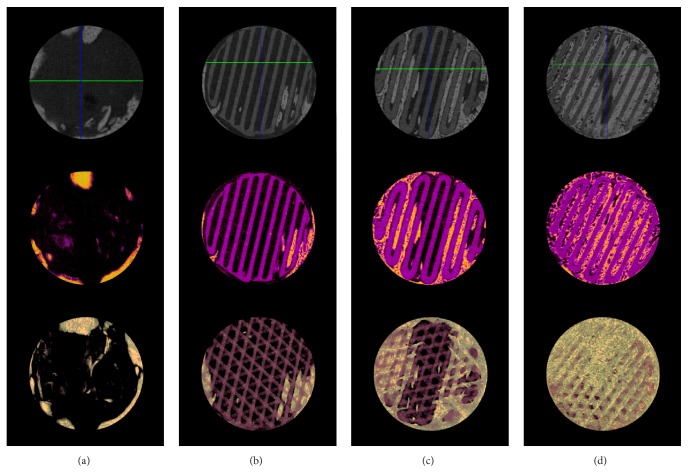
Microcomputed tomographic (*μ*CT) images of the four study groups. (a) Control group, (b) PCL/*β*-TCP group, (c) PCL/*β*-TCP/bdECM group, (d) and PCL/*β*-TCP/bdECM/BMP group. In the second row of color images, scaffolds are purple and new bone is yellow. The third row shows reconstructed images.

**Figure 9:**
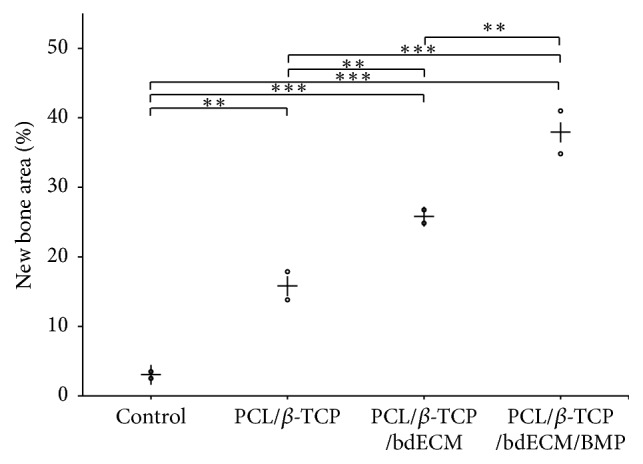
Scatter plots and medians (represented by crosses) of new bone volume (mm^3^) (^*∗∗*^*p* < .01 and ^*∗∗∗*^*p* < .001).

Volumetric measurement results obtained by *μ*CT are shown in Table 1 and Figures 8 and 9. At 4 weeks, average mean (±SD) new bone volumes (mm^3^) in the control, PCL/*β*-TCP, PCL/*β*-TCP/bdECM, and PCL/*β*-TCP/bdECM/BMP groups were 2.98 (±0.68), 15.83 (±2.86), 25.79 (±1.36), and 37.88 (±4.36), respectively, and intergroup differences were significant (*p* < .001). New bone volume (mm^3^) was significantly higher in all experimental groups than in the control group at week 4 (*p* < .01). The PCL/*β*-TCP/bdECM/BMP group showed the highest new bone volume (mm^3^) and the control group the lowest.

#### 3.2.3. Histological Findings

**Figure 10:**
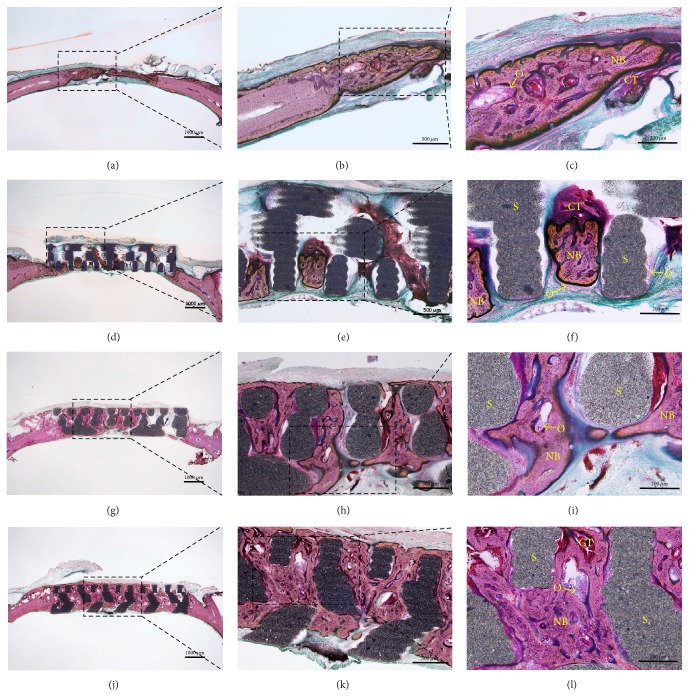
Hematoxylin-eosin (H&E) stained sections at 4 weeks after surgery. Control group (a, b, and c), PCL/*β*-TCP group (d, e, and f), PCL/*β*-TCP/bdECM group (g, h, and i), and PCL/*β*-TCP/bdECM/BMP group (j, k, and l). NB: new bone, CT: connective tissue, O: osteoblasts, and S: 3D-printed scaffold [original magnifications: ×12.5 (a, d, g, and j), ×40 (b, e, h, and k), and ×100 (c, f, i, and l)].

Histological findings are shown in Figure 10. Abnormal findings, such as inflammation, were not observed in any group, and grafted scaffolds were well positioned in calvarial defects. At 4 weeks after surgery, calcification and new bone formation were observed in all four groups. In control group, fibrous and connective tissues were observed as layers in calvarial defects, while, in the PCL/*β*-TCP group, fibrous and connective tissues were observed in scaffolds and small amounts of new bone originating from old bone were observed. More new bone formation was observed in the PCL/*β*-TCP/bdECM and PCL/*β*-TCP/bdECM/BMP groups than in the control and PCL/*β*-TCP groups. In particular, interstitial spaces in the PCL/*β*-TCP/bdECM/BMP group had been filled by new bone and fibrous and connective tissues.

#### 3.2.4. Histometric Findings

**Figure 11:**
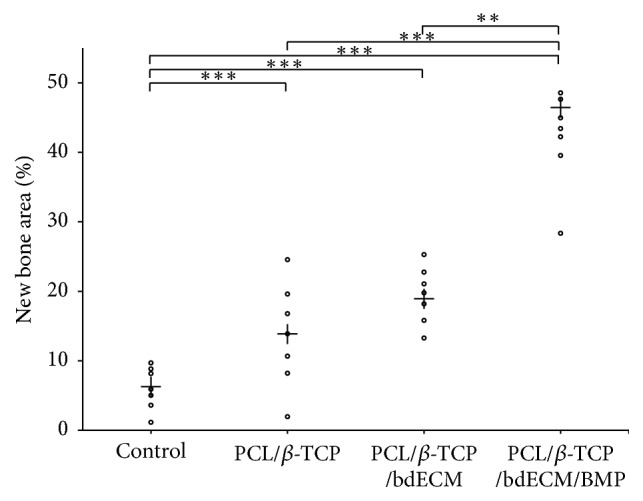
Scatter plots and median values (crosses) for new bone areas at 4 weeks after surgery (%) (^*∗∗*^*p* < .01 and ^*∗∗∗*^*p* < .001).

Histometric results are summarized in Table 2 and Figure 11. At 4 weeks after surgery, new bone areas (%) in the control, PCL/*β*-TCP, PCL/*β*-TCP/bdECM, and PCL/*β*-TCP/bdECM/BMP groups were 6.12 (±3.64), 13.65 (±7.52), 19.17 (±4.42), and 43.32 (±7.63), respectively, and new bone area (%) values were significant between groups (*p* < .01). New bone area (%) was significantly greater in the three experimental groups than in the control group (*p* < .01). New bone area (%) was highest in the PCL/*β*-TCP/bdECM/BMP group and lowest in the control group. However, no significant difference was observed between the PCL/*β*-TCP/bdECM and PCL/*β*-TCP groups (*p* > .05).

## 4. Discussion

In the tissue engineering field, solid-free form fabrication (SFF) technology has been performed using techniques, such as selective laser sintering (SLS), stereolithography (SLA), fused deposition modeling (FDM), and multihead deposition systems (MHDS) [4]. MHDS have four heads of temperature and pressure control parts and can produce 3D structures quickly using different biomaterials using a layering process [29]. In addition, MHDS do not require toxic solvents and thus do not adversely affect cells [29]. In the present study, MHDS fabricated biodegradable scaffolds were designed to allow cells to reach the centers of scaffolds through interconnected triangular pores. In biodegradable scaffolds, porous microstructure importantly regulates mechanical functions and bone regeneration. The interconnected scaffold pores facilitate oxygen, waste, and nutrient transport [6]. Pores size has been shown to be associated with bone regeneration, and some studies have reported that larger pore sizes increase the differentiation and proliferation of osteoblasts [30, 31]. Adachi et al. [30] compared two groups with different pore sizes (100–300 *μ*m and 500–700 *μ*m) and reported excellent bone differentiation results in the 500–700 *μ*m group. In the present study, the scaffolds used had a diameter of 8 mm and when pore size was set to ≥500 *μ*m, printing accuracy and mechanical strengths decreased and, thus, we used a constant pore size of 400 *μ*m. The advantage of 3D printing is that it can be used for its ability to produce different structures and, therefore, it presents a means of optimizing internal structures to further enhance bone formation.

Blood should penetrate scaffolds easily, but this penetration and cell adherence are inhibited by the hydrophobic natures of synthetic polymers like PCL and, thus, we used *β*-TCP (a highly hydrophilic bioceramic) to address this problem [32, 33]. *β*-TCP is known to promote osteoblast formation and is easily absorbed by osteoclasts and macrophages [32]. In addition, *β*-TCP releases calcium ions that promote bone differentiation and, thus, higher *β*-TCP contents would be expected to promote bone differentiation [34]. When the percentage of *β*-TCP in PCL is increased, viscosity also increases, and as PCL/*β*-TCP blend viscosity affects scaffold printing speed, 3D printer feed rate reduces and the hydrogel is exposed to more thermal energy. While fabricating scaffolds use different feed viscosities, we observed that scaffold mechanical strength and printer feed rate were reduced by as much as 60%. In this study, the PCL/*β*-TCP ratio was set at 70 : 30 (wt%) to achieve balance between *β*-TCP content and printing rate.

The effect of bone growth factor rhBMP-2 depends on the type of carrier [26], which includes materials like collagen [35], fibrin [36], and hyaluronic acid [37], and optimal bone formation occurs when rhBMP-2 is released in a consistent manner. In the majority of previous studies on the sustained release of rhBMP-2, initial burst release was reported before sustained release was achieved [27, 38, 39]. In our study of the release kinetics of rhBMP-2 from PCL/*β*-TCP/bdECM/BMP, rhBMP-2 release was found to be more consistent, indicating the suitability of bdECM as a rhBMP-2 carrier.

When the 3D printed scaffold is grafted in the bony defect, it first encounters cells, and since scaffold affects cellular activities, such as cell attachment, differentiation, and death, therefore scaffold should have a good cell bioactivity. In the present study, higher initial cell adhesion was observed for samples containing bdECM, which suggests that additional cell binding sites provided by bdECM enhance cell adhesion rates. In addition, as a result of ALP expression which regarded early marker of osteogenesis and amount of calcium deposition, PCL/*β*-TCP/bdECM/BMP group showed the highest ALP expression. This result indicates that printed bdECM containing rhBMP-2 promotes osteogenic differentiation. In our animal study, defect areas in the PCL/*β*-TCP/bdECM/BMP group were successfully filled with new bone, and the PCL/*β*-TCP/bdECM/BMP scaffold was found to improve bone regeneration as compared with other scaffolds. The well-formed interconnected pores of scaffolds enable cell migration easier, and the cell contacts the biomolecules of the bdECM promoting cell adhesion to the scaffold [6, 40]. In addition, rhBMP-2 released from bdECM would be expected to promote osteogenic differentiation [7, 41].

La et al. coated bdECM onto 3D printed scaffolds and observed significantly more new bone formation than in an uncoated group in a mouse calvaria defect model [18]. In contrast, in the present study, histometric findings were similar in PCL/*β*-TCP and PCL/*β*-TCP/bdECM groups. This difference seems to be because La et al. used a dip coating method which enables coating bdECM more widely onto scaffolds, whereas we printed bdECM between PCL/*β*-TCP lines using a 3D printer.

It is considered that using the dip coating method for bdECM is more widely applied to the bdECM on scaffold than the printing method. Within the limitations of this study, treatment of calvarial defects with 3D printed PCL/*β*-TCP/bdECM/BMP scaffolds significantly enhanced new bone formation. However, our in vivo observations were made at 4 weeks after implantation using bdECM at a single concentration and one application method. Therefore, we suggest further longer-term studies to be conducted to optimize application conditions.

## 5. Conclusions

The PCL/*β*-TCP/bdECM/BMP scaffold of this study was produced by printing bdECM containing rhBMP-2 between PCL/*β*-TCP lines using 3D printing technology. bdECM stably carried rhBMP-2 and was found to enhance cell adhesion and promote osteogenic differentiation. Furthermore, in the rat calvarial defect model, no side effects, such as immune response, were observed and excellent bone regeneration was obtained.

## Figures and Tables

**Table 1:** New bone volumes within regions of interest (*n* = 2; mm^3^).

Group	Mean ± SD	Median
Control	2.98 ± 0.68	2.98
PCL/*β*-TCP	15.83 ± 2.86	15.83
PCL/*β*-TCP/bdECM	25.79 ± 1.36	25.79
PCL/*β*-TCP/bdECM/BMP	37.88 ± 4.36	37.88

*p* value	<.001	

**Table 2:** New bone areas within areas of interest (*n* = 7, %).

Group	Mean ± SD	Median
Control	6.12 ± 3.64	6.23
PCL/*β*-TCP	13.65 ± 7.52	13.87
PCL/*β*-TCP/bdECM	19.17 ± 4.42	18.97
PCL/*β*-TCP/bdECM/BMP	43.32 ± 7.63	46.24

*p* value	<.001	
